# Primary cilia suppress the fibrotic activity of atrial fibroblasts from patients with atrial fibrillation in vitro

**DOI:** 10.1038/s41598-024-60298-x

**Published:** 2024-05-30

**Authors:** Makiri Kawasaki, Rushd F. M. Al-Shama, Fransisca A. Nariswari, Benedetta Fabrizi, Nicoline W. E. van den Berg, Robin Wesselink, Jolien Neefs, Eva R. Meulendijks, Sarah W. E. Baalman, Antoine H. G. Driessen, Joris R. de Groot

**Affiliations:** Amsterdam UMC, Department of Clinical and Experimental Cardiology, Amsterdam Cardiovascular Sciences, Heart Center, University of Amsterdam, Meibergdreef 9, 1105 AZ Amsterdam, The Netherlands

**Keywords:** Cell biology, Molecular biology

## Abstract

Atrial fibrosis serves as an arrhythmogenic substrate in atrial fibrillation (AF) and contributes to AF persistence. Treating atrial fibrosis is challenging because atrial fibroblast activity is multifactorial. We hypothesized that the primary cilium regulates the profibrotic response of AF atrial fibroblasts, and explored therapeutic potentials of targeting primary cilia to treat fibrosis in AF. We included 25 patients without AF (non-AF) and 26 persistent AF patients (AF). Immunohistochemistry using a subset of the patients (non-AF: n = 10, AF: n = 10) showed less ciliated fibroblasts in AF versus non-AF. Acetylated α-tubulin protein levels were decreased in AF, while the gene expressions of *AURKA* and *NEDD9* were highly increased in AF patients’ left atrium. Loss of primary cilia in human atrial fibroblasts through *IFT88* knockdown enhanced expression of ECM genes, including *FN1* and *COL1A1*. Remarkably, restoration or elongation of primary cilia by an AURKA selective inhibitor or lithium chloride, respectively, prevented the increased expression of ECM genes induced by different profibrotic cytokines in atrial fibroblasts of AF patients. Our data reveal a novel mechanism underlying fibrotic substrate formation via primary cilia loss in AF atrial fibroblasts and suggest a therapeutic potential for abrogating atrial fibrosis by restoring primary cilia.

## Introduction

Atrial fibrillation (AF) is the most common sustained arrhythmia associated with a five-fold increased risk of stroke^1,2^ and heart failure^3^, and has been associated with a substantial deterioration of patients’ quality of life^4^. AF is an independent risk factor of all-cause mortality, increasing it by 1.5–2 fold^5^. The prevalence of AF is projected to increase in the next decades^6^. In 2010, the number of patients suffering from AF was estimated at approximately 8.8 million in the European Union alone^7^ and 33.5 million worldwide^8^. Antiarrhythmic drugs (AADs) and catheter or surgical ablation may be employed to restore sinus rhythm, but these remain modestly successful^9–12^.

Atrial fibrosis promotes both ectopy and conduction abnormalities^13,14^ and serves as an essential substrate that sustains and perpetuates AF. As a result, atrial fibrosis is a predictor of poor outcomes of ablative and pharmacologic rhythm control strategies for AF^15–17^. At the moment, attempts to prevent or reduce atrial fibrosis by interfering with profibrotic pathways, such as the renin–angiotensin–aldosterone system using angiotensin-converting enzyme inhibitors or angiotensin receptor blockers, have been only partially effective^18,19^. An explanation of the limited efficacy of current strategies to treat fibrosis may lie in targeting a specific profibrotic pathway rather than targeting the process of fibrosis formation as a whole.

Atrial fibrosis results from the aberrant accumulation of extracellular matrix (ECM). ECM is a highly dynamic structure that is constantly remodeled through the deposition of ECM proteins such as collagens and degradation by matrix metalloproteases^20^. Fibroblast activation is key in both processes. A dynamic ECM balanced by the activities of fibroblasts is necessary for tissue plasticity and integrity^20^. However, in a pathological condition such as AF, fibroblasts undergo increased proliferation and differentiation into α-smooth muscle actin (αSMA)-expressing myofibroblasts^21^, which causes discord in ECM dynamics, ultimately culminating in interstitial fibrosis within the atria. Targeting fibroblast activation is challenging because it can be induced by various stimuli within intricate biological processes involving a large number of proteins^22^. Importantly, our knowledge of what drives fibroblasts to translate the wide variety of stimuli into an exaggerated fibrotic response in AF is limited.

**Figure 1:**
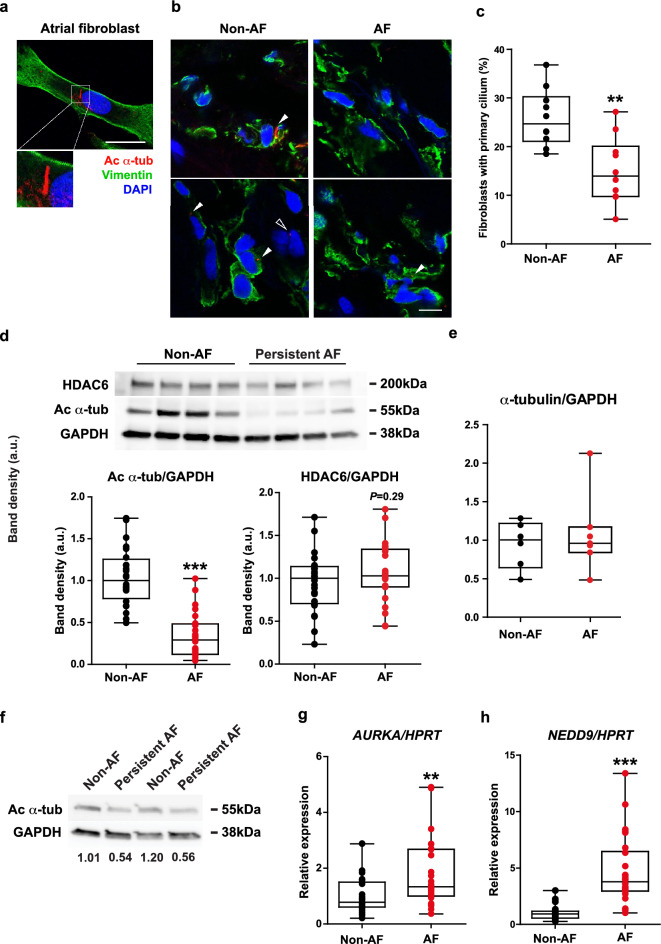
The proportion of fibroblasts with primary cilia is significantly decreased in the left atrial tissue of AF patients possibly via the NEDD9/AURKA/HDAC6 axis. (**a**) A representative morphology of primary cilium projecting from the apical surface of a cultured fibroblast isolated from the left atrial tissue of a non-AF patient. Red: ac α-tub (acetylated α-tubulin, primary cilium), green: vimentin (intermediate cytoskeleton), blue: DAPI (nucleus). Fibroblasts were starved for 48 h to promote the formation of primary cilia. Scale bar, 25 μm. **p < 0.01 (Student’s unpaired *t*-test). (**b**) Representative images of immunohistochemistry performed on the cryosections of left atrial tissue from the non-AF and AF patients. Arrow heads and a void arrowhead indicate the primary cilia of vimentin positive and vimentin negative cells, respectively. Scale bar, 10 μm. (**c**) The proportion of fibroblasts with primary cilia (%) in the left atrial tissue of non-AF and AF patients. Approximately 300–500 cells/patient were counted. **p < 0.01 (Student’s unpaired *t*-test). (**d**) Protein levels of ac α-tub and HDAC6 in the left atrial tissue of non-AF and AF patients. GAPDH serves as a loading control. **p < 0.01 (Mann–Whitney U test). (**e**) The protein levels of α-tubulin in the left atrial tissue of non-AF and AF patients. N = 6/group. (**f**) The protein levels of ac α-tub in the fibroblast fraction isolated from left atrial tissue of non-AF and AF patients. The values at the lowest row show the band density of ac α-tub relative to GAPDH in each lane. (**g**, **h**) The gene expression of *AURKA* (**g**) and *NEDD9* (**h**) in the left atrial tissue of non-AF and AF patients. *HPRT* serves as an internal control. **p < 0.01, ***p < 0.001 (Mann–Whitney U test).

The primary cilium is a cellular sensory organelle composed of acetylated microtubules that exists in nearly all mammalian cells, including human atrial fibroblasts (Fig. 1a, white box)^23,24^. It protrudes from the cellular membrane into the extracellular space and functions as a signaling hub that transduces external mechanical and chemical cues into intracellular signals^25–28^. The primary cilium appears only at the G_0_ phase^29,30^, whereas its assembly is systematically restricted during the growth phase^23,30^. This dynamic morphology qualifies the primary cilium to serve as a spatiotemporal sensor that fine-tunes transduction from the external milieu into the cell^31^. Accordingly, loss of the primary cilium induces abnormal proliferation^24^ and affects various signal transduction, including renin-angiotensin^32^ and TGF-β1 signaling^33^, both of which are crucial for fibrosis formation in AF^34^. Genetic mutations affecting primary cilia induce fibrosis in multiple organs, including the liver and kidneys^35^. Furthermore, aberrant shortening of the primary cilium has been linked to age-related acquired diseases, such as cancers^36^ and atherosclerosis^37^.

Key proteins regulate ciliary formation and assembly. The interaction between aurora kinase A (AURKA) and neural precursor cell expressed developmentally down-regulated protein 9 (NEDD9) phosphorylates and activates histone deacetylase 6 (HDAC6)^38^. HDAC6 then deacetylases acetylated α-tubulin, leading to a rapid collapse of ciliary axoneme^38,39^. This leads to primary cilia disassembly. A concomitant increase in HDAC6 activity and reduction in acetylated α-tubulin was indeed reported in the left atrial tissue of AF patients^40^. Furthermore, our recent transcriptomic study performed on the left atrial tissue from patients with or without AF revealed that genes relevant to primary cilia assembly were downregulated (e.g., a number of intraflagellar transport genes) while disassembly genes were upregulated (e.g., *NEDD9* and *LIMK2*) in AF compared to non-AF patients^41^. Conversely, the presence of primary cilia in cardiac fibroblasts was suggested to contribute to fibrosis formation in infarcted myocardium^42^.

We hypothesized that the disassembly of primary cilia in fibroblasts is the central link between external profibrotic stimuli and the formation of fibrosis in AF. Therefore, we examined the function of primary cilia in respect to the profibrotic capacity of atrial fibroblasts from patients with AF.

## Results

### Loss of primary cilia is observed in the atrial tissue of AF patients

In this study, we included patients with persistent AF^43^ (AF: n = 26) or without a history of AF^44,45^ (non-AF: n = 25) (Table 1). The tissue from a subgroup of 10 AF patients and 10 non-AF patients was used for immunohistochemical analysis (Supplemental Table 1). The two cohorts were matched in terms of gender and BMI. AF patients were relatively younger than non-AF patients (65.8 ± 9.1 vs. 70.8 ± 7.5, *p* = 0.04) (Table 1) in the entire cohort, but the age of subgroup cohorts for immunohistochemical analysis was matched (69.2 ± 6.6 vs. 72.5 ± 6.4, *p* = 0.27) (Supplemental Table 1). The major differences between the AF and non-AF cohorts were the presence of vascular disease and medication (Table 1 and Supplemental Table 1).

**Table 1:** Clinical characteristics of the patients enrolled in this study.

	Non-AF (n = 25)	Persistent AF (n = 26)	*p*-value
Surgery type			
VATS PVI	–	26 (100)	*NA*
CABG	22 (88)	–	*NA*
Aortic valve	5 (20)	–	*NA*
CABG+valve	4 (16)	–	*NA*
Bentall	2 (8)	–	*NA*
Baseline			
Sex, male, n(%)	18 (72)	19 (73)	1
Age, years (± s.d.)	70.8 ± 7.5	65.8 ± 9.1	0.04
AF duration, years [IQ]	–	3.0 [1.3–5.0]	*NA*
Previous catheter PVI, n(%)	–	2 (8)	*NA*
BMI, kg/m^2^ (± s.d.)	27.8 ± 2.5	28.1 ± 4.2	0.753
Creatinine, μl/l (± s.d.)	85.0 ± 24.6	87.5 ± 22.2	0.72
CHA2DS2-VASc [IQ]	3 [2-4]	1 [1-2]	< 0.001
Vascular disease, n(%)	22 (88)	2 (8)	< 0.001
Previous PCI, n(%)	6 (24)	0 (0)	0.026
Myocardial infarction, n(%)	9 (36)	1 (4)	0.011
Hypertension, n(%)	15 (60)	12 (46)	0.478
Diabetes Mellitus, n(%)	5 (20)	2 (8)	0.384
Stroke/TIA/embolus, n(%)	5 (20)	3 (12)	0.656
Medication			
NOAC/vitK antagonist, n(%)	0 (0)	26 (100)	< 0.001
Antiplatelet, n(%)	22 (88)	1 (4)	< 0.001
Class IA AAD, n(%)	0 (0)	1 (4)	1
Class IC AAD, n(%)	0 (0)	7 (27)	0.017
Class II AAD, n(%)	19 (76)	11 (42)	0.031
Class III AAD, n(%)	1 (4)	8 (31)	0.032
Class IV AAD, n(%)	0 (0)	4 (15)	0.128
Digoxin, n(%)	0 (0)	5 (19)	0.066
ACE inhibitor, n(%)	9 (36)	7 (27)	0.692
Angiotensin II blocker, n(%)	5 (20)	6 (23)	1

VATS PVI, video assisted thoracoscopic pulmonary vein isolation; CABG, coronary artery bypass grafting; BMI, body mass index; PCI, percutaneous coronary intervention; TIA, transient ischemic attack; NOAC, non-vitamin K antagonist oral anticoagulants;AAD, antiarrhythmic drugs. The categorical variables (eg. sex, CHA2DS2-VASc, the presence of comorbidities and medication) were compared by Chi-squared test. The continuous variables (eg. age, BMI and creatinine) were compared by Mann–Whitney U test.

Immunostaining of acetylated α-tubulin performed on the subgroup demonstrated a lower proportion of vimentin-positive cells (which are mostly fibroblasts) with the signal of acetylated α-tubulin in the left atrial tissue of AF compared to non-AF patients (Fig. 1b,c). Consistent with this observation, the protein levels of acetylated α-tubulin were dramatically decreased in the left atrial tissue of AF patients compared to non-AF patients (Fig. 1d). The total α-tubulin protein levels were equal between non-AF and AF patients (Fig. 1e). This, in combination with the markedly decreased levels of acetylated α-tubulin, indicates that the acetylation levels but not expression levels of α-tubulin were decreased in AF patients. Importantly, the protein levels of acetylated α-tubulin were also reduced in the fibroblast fraction isolated from left atrial tissue of AF patients compared to non-AF patients (Fig. 1f), suggesting that the loss of primary cilia may occur primarily in the fibroblast fraction. The proportion of vimentin-negative cells (mostly cardiomyocytes) with primary cilia did not significantly differ between the groups (Supplemental Fig. 1c).

### The activation of NEDD9/AURKA/HDAC6 in AF is potentially responsible for the loss of cilia

The protein levels of HDAC6 were equivalent between AF and non-AF patients (Fig. 1d). However, the gene expression of *AURKA* and *NEDD9* was significantly upregulated in AF (Fig. 1e,f), suggesting that the activity of HDAC6 is enhanced in AF as was previously reported^40^. Pitchfork (PIFO) and trichoplein keratin binding (TCHP) are also reported to activate AURKA^24,46^, but their gene expression was barely altered between AF and non-AF (Supplemental Fig. 1d,e). These results indicate that loss of the primary cilia in the atrial fibroblasts may be induced by the activated NEDD9/AURKA/HDAC6 axis in AF.

### Loss of the primary cilia increases the profibrotic capacity of human atrial fibroblasts

**Figure 2:**
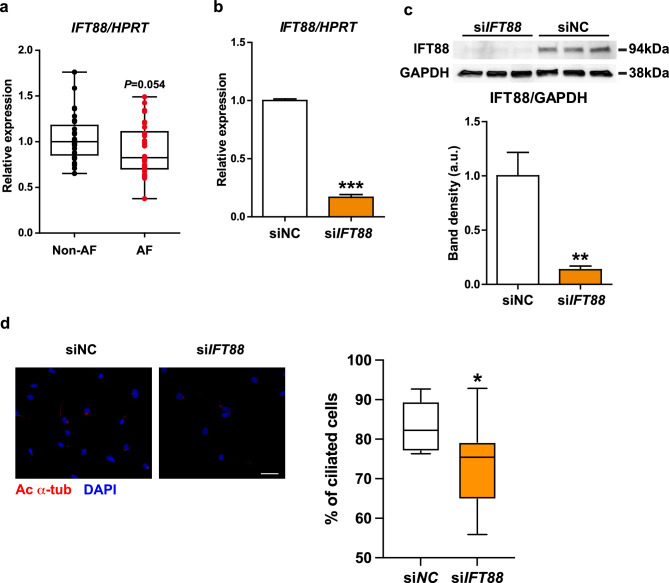
Loss of the primary cilia through knockdown of *IFT88* reduces the proportion of NHCF-A cells with primary cilia. (**a**) The gene expression of *IFT88* in the left atrial tissue of non-AF and AF patients tested by Mann–Whitney U test. (**b**, **c**) The gene expression (**b**) and the protein levels (**c**) of IFT88 in NHCF-A cells transfected with siNC (negative control) or si*IFT88*. Error bars, mean (SD). N = 3/group. **p < 0.01, ***p < 0.001 (Student’s unpaired *t*-test). (**d**) The representative images and quantification of ciliated cells (%) in NHCF-A cells transfected with siNC or si*IFT88*. Red: acetylated α-tubulin (primary cilia), blue: DAPI (nuclei). Scale bar, 50 μm. *p < 0.05 (Mann–Whitney U test); n = 4/group (101–122 cells/replicate).

Next, we examined the consequences of loss of the primary cilia in human atrial fibroblasts. We observed a trend toward a decrease in gene expression of intraflagellar transport homolog 88 (*IFT88*), an essential factor for the formation of primary cilia^47,48^ in the left atrial tissue of AF patients compared to non-AF patients (Fig. 2a). Therefore, we silenced the *IFT88* gene by RNAi in primary human atrial fibroblasts isolated from normal adult atrial tissue (hereafter, NHCF-A cells). We observed substantial knockdown efficiency of siRNA targeting *IFT88* both at gene and protein levels (Fig. 2b,c). The knockdown of *IFT88* significantly decreased the proportion of cells with primary cilia (Fig. 2d). The structure of the primary cilia without IFT88 knockdown in these cells is shown in Supplemental Fig. 1a,b.

**Figure 3:**
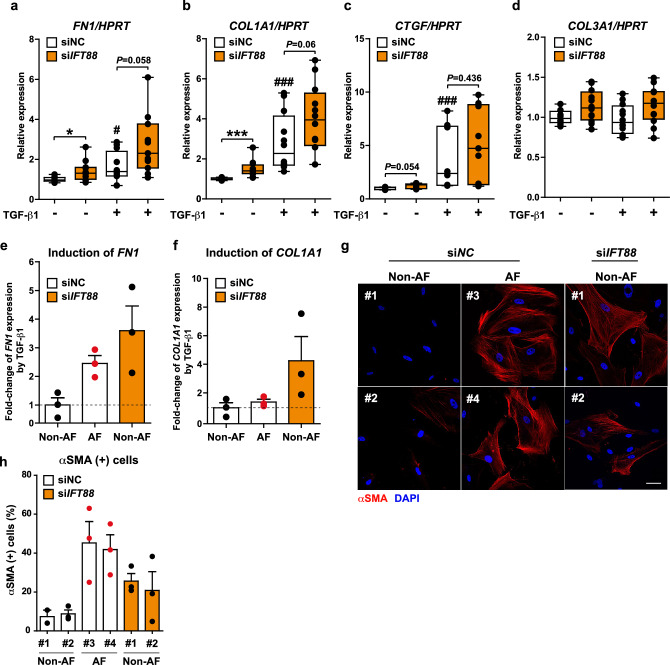
Loss of the primary cilia through knockdown of IFT88 enhances the profibrotic capacity of NHCF-A cells and the atrial fibroblasts isolated from non-AF patients. (**a**–**d**) The gene expression of *FN1* (**a**), *COL1A1* (**b**), *CTGF* (**c**), and *COL3A1* (**d**) in the NHCF-A cells transfected with siNC (negative control) or si*IFT88* and cultured in the absence (vehicle) or presence of 2 ng/mL TGF-β1 for 48 h. *HPRT* serves as an internal control. ^#^*p* < 0.05, ^###^*p* < 0.001 vs. siNC group treated with vehicle, *p < 0.05, ***p < 0.001 (Student’s unpaired *t*-test or Mann–Whitney U test were employed according to the data distribution, and all the *p*-values were adjusted by post-hoc analysis). (**e**, **f**) The induction of *FN1* (**e**) and *COL1A1* (**f**) gene expression by TGF-β1 in the fibroblasts isolated from the left atrial tissue of three non-AF and three AF patients is shown as a fold-change (TGF-β1 treated/vehicle treated group). The dashed line indicates the baseline. (**g**, **h**) The representative images (**g**) and quantification (**h**) of αSMA-expressing myofibroblasts isolated from two non-AF (#1 and #2) and two AF patients (#3 and #4). Fibroblasts from non-AF patients were transfected with siNC or si*IFT88*, and fibroblasts from AF patients were transfected with siNC. Red: αSMA (myofibroblasts), blue: DAPI (nuclei). Scale bar, 50μm. The proportion of αSMA-positive cells was quantified from three technical replicates. Error bar, mean (SD).

The gene expression of ECM components such as fibronectin-1 *(FN1)*, alpha-1 type I collagen *(COL1A1)*, and connective tissue growth factor (*CTGF*) demonstrated a considerable or trending toward up-regulation in *IFT88*-silenced NHCF-A cells both in the presence or absence of TGF-β1 (Fig. 3a–c), suggesting that loss of the primary cilia in atrial fibroblasts enhances ECM production. The gene expression of alpha-1 type III collagen (*COL3A1*) remained unchanged (Fig. 3d).

Next, we silenced *IFT88* in the atrial fibroblasts isolated from non-AF patients (hereafter, non-AF fibroblasts) to examine if the knockdown of *IFT88* in non-AF fibroblasts induces a phenotype switch to atrial fibroblasts isolated from AF patients (hereafter, AF atrial fibroblasts). As expected, when *IFT88* was silenced in non-AF fibroblasts, the gene expression of *FN1* and *COL1A1* was induced by TGF-β1 at similar to or even higher levels than AF atrial fibroblasts (Fig. 3e,f). The capacity of non-AF fibroblasts to differentiate into myofibroblasts was also enhanced when *IFT88* was silenced, although the degree was slightly less than that of AF fibroblasts (Fig. 3g,h). Taken together, our results imply that loss of the primary cilia observed in AF patients induces an enhanced differentiation of atrial fibroblasts into myofibroblasts and increases the expression of genes that are components of ECM.

### Restoration of the primary cilium blunts the profibrotic response of human atrial fibroblasts

**Figure 4:**
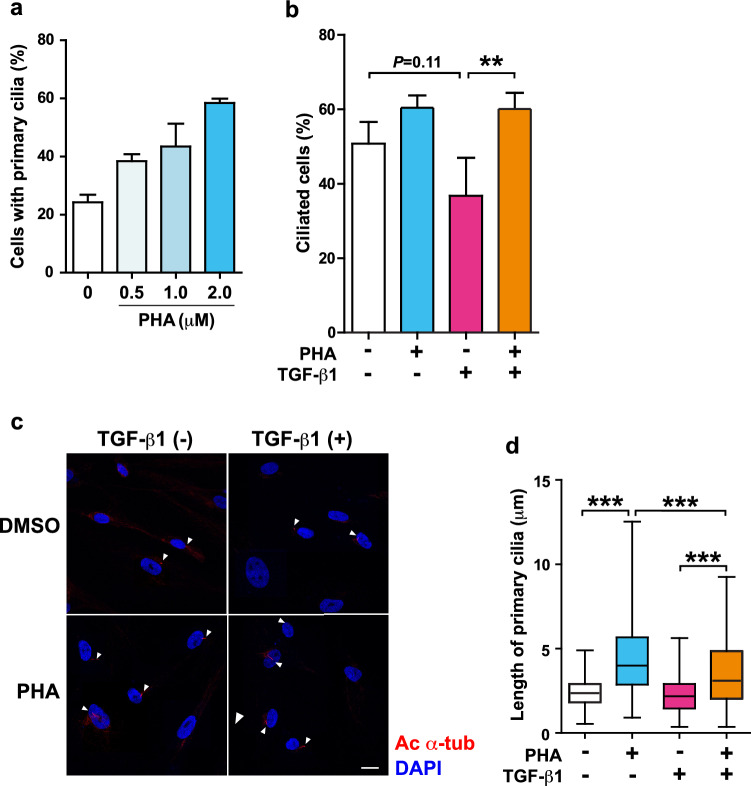
PHA promotes the cilia formation and increase the length of primary cilia in NHCF-A cells. (**a**) The proportion of NHCF-A cells with primary cilia cultured in the growth medium supplemented with 10%FBS, hFGF-B and insulin following a PHA treatment at different doses (0, 0.5, 1.0 or 2.0 μM) for 48 h. N = 2/treatment. Error bars, mean (SD). (**b**–**d**) The proportion of ciliated NHCF-A cells (**b**), the representative morphology (**c**) and the length of primary cilia (**d**) when NHCF-A cells were cultured in 0.5% FBS medium with vehicle or 2 ng/mL TGF-β1 in the absence (DMSO) or presence of 2 μg/mL PHA. Arrow heads indicate primary cilia. Red: ac α-tub (primary cilia), blue: DAPI (nuclei). Scale bar, 20 μm. In total, > 100 primary cilia/treatment from three technical replicates were measured. Error bars, mean (SD). **p < 0.01 in (**b**) (Student’s unpaired *t*-test). ***p < 0.001 in (**c**) (Mann–Whitney U test).

To explore the effects of protecting primary cilia from disassembly on the activation of atrial fibroblasts, we inhibited the NEDD9/AURKA/HDAC6 axis with PHA-680632 (PHA), a selective inhibitor of AURKA. PHA dose-dependently restored the primary cilia in NHCF-A cells cultured in the growth medium (supplemented with 10% FBS, hFGF-B, and insulin), where the formation of primary cilia is systematically restricted (Fig. 4a). On the other hand, in the medium supplemented with 0.5% FBS, where many cells exit from the cell cycle, PHA treatment didn’t dramatically increase ciliated NHCF-A cells (%) (Fig. 4b, the left two bars). However, it counteracted the decreased tendency of ciliated NHCF-A cells (%) in TGF-β1 treatment (Fig. 4b, the right two bars). Furthermore, PHA significantly increased the length of primary cilia both in the presence and absence of TGF-β1. However, its effect was partially but significantly inhibited by TGF-β1 (Fig. 4c,d).

**Figure 5:**
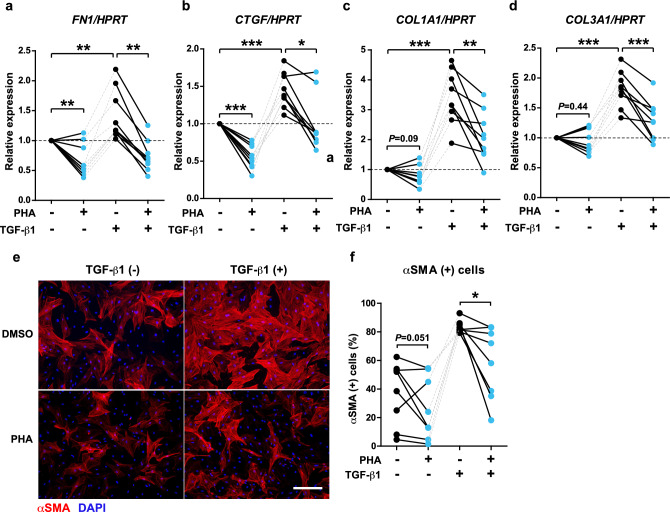
PHA (AURKA selective inhibitor PHA-680632) inhibits the profibrotic response of the fibroblasts isolated from the left atrial tissue of AF patients. (**a**–**d**) The gene expression of *FN1* (**a**), *COL1A1* (**b**), *CTGF* (**c**) and *COL3A1* (**d**) in the fibroblasts isolated from AF patients and cultured with vehicle or 2 ng/mL TGF-β1 in the absence (DMSO) or presence of 2 μg/mL PHA. The dashed line indicates the baseline. ^##^*p* < 0.01, ^###^*p* < 0.001 vs. PHA (−) TGF-β1 (−) group, *p < 0.05, **p < 0.01, ***p < 0.001 (paired *t*-test or Wilcoxon signed-rank test according to the data distribution). (**e**, **f**) The representative images (**e**) and the quantification (**f**) of fibroblasts from AF patients that differentiated into αSMA-expressing myofibroblasts in respective treatments. Red: αSMA (myofibroblasts), blue: DAPI (nuclei). Scale bar, 100 μm. *p < 0.05 (paired *t*-test).

Next, we attempted to abrogate the profibrotic response of AF fibroblasts isolated from AF patients by restoring their primary cilia using PHA. In atrial fibroblasts isolated from the left atrial tissue of AF patients, the expression of ECM genes at baseline and in the presence of TGF-β1 was dramatically suppressed in the presence of PHA (Fig. 5a–d). This effect could be partly due to a suppression of fibroblast differentiation into myofibroblasts, as the proportion of αSMA-positive cells was notably lower when the cells were treated with TGF-β1 in the presence of PHA (Fig. 5e,f).

**Figure 6:**
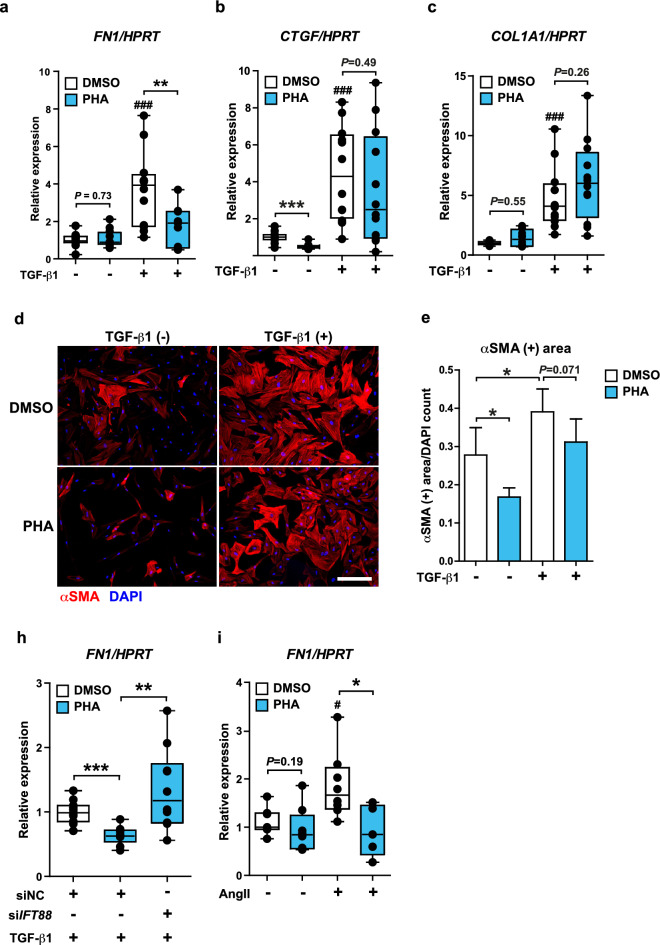
PHA suppresses the gene expression of *FN1* induced by TGF-β1 in a primary cilia-dependent manner. (**a**–**c**) The gene expression of *FN1* (**a**), COL1A1 (**b**), CTGF (**c**) in NHCF-A cells when cells were cultured with vehicle or 2 ng/mL TGF-β1 for 48 h in the absence (DMSO) or presence of 2 μM PHA in respective treatments. ^###^*p* < 0.001 vs. PHA (−) TGF-β1 (−) group, **p < 0.01 (Mann–Whitney U test). (**d**, **e**) The representative images of NHCF-A cells that differentiated into αSMA positive myofibroblasts (**d**) and the quantification of αSMA positive area (**e**). Red: αSMA (myofibroblasts), blue: DAPI (nuclei). Scale bar, 100 μm. αSMA positive area is normalized by DAPI count (a.u.). N = 5/group. Error bars, mean (SD). *p < 0.05 (Student’s unpaired *t*-test). (**h**) The gene expression of *FN1* when NHCF-A cells were first transfected with siNC or si*IFT88* and then cultured with 2 ng/mL TGF-β1 in the absence (DMSO) or presence of 2 μg/mL PHA. **p < 0.01, ***p < 0.001 (Student’s unpaired *t*-test). (**i**) The gene expression of *FN1* in NHCF-A treated with vehicle or 10^−6^ M AngII (angiotensin II) for 48 h in the absence of presence of 2 μg/mL PHA. ^#^*p* < 0.05, vs. PHA (−) AngII (−) group, *p < 0.05 (Mann–Whitney U test).

In NHCF-A cells, PHA suppressed the gene expression of *FN1* (Fig. 6a) but not of *CTGF* and *COL1A1* in the presence of TGF-β1 (Fig. 6b,c). Consistently, the differentiation of NHCF-A cells into myofibroblasts was incompletely inhibited (Fig. 6d,e). The limited efficacy of PHA on NHCF-A from a healthy donor suggests that PHA may be more effective in atrial fibroblasts from AF patients due to their supraphysiological activity elicited in the diseased status. Importantly, the inhibitory effect of PHA on *FN1* gene induction by TGF-β1 was completely abolished when the formation of primary cilia was inhibited by si*IFT88* in NHCF-A cells (Fig. 6h). Thus, the mechanism of action of PHA appears to depend on primary cilia. Remarkably, PHA also suppressed *FN1* gene induction by angiotensin II (AngII), another important inducer of atrial fibrosis in AF^49^ (Fig. 6i), indicating that PHA is potentially capable of suppressing the profibrotic reaction of atrial fibroblasts in response to multiple profibrotic stimuli.

**Figure 7:**
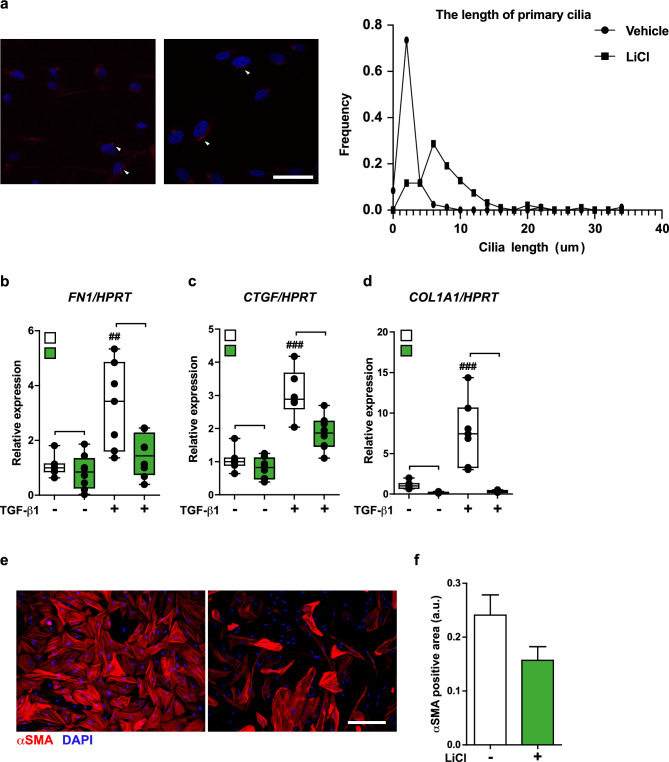
LiCl elongates primary cilia and inhibits the profibrotic response of the NHCF-A and dedifferentiates them into αSMA-negative cells. (**a**) The representative images and the quantification of cilia length with LiCl treatment. Red: acetylated α-tubulin (primary cilia), blue: DAPI (nuclei). Scale bar, 20 μm *p < 0.05 (Mann–Whitney U test); n = 4/group (20–29 cells/sample). (**b**–**d**) The gene expression of *FN1* (**b**), *CTGF* (**c**) and *COL1A1* (**d**) when NHCF-A cells were cultured with vehicle or 2 ng/mL TGF-β1 for 48 h in the absence (water) or presence of 50 mM LiCl. ^##^*p* < 0.01, ^###^*p* < 0.001 vs. LiCl (−) TGF-β1 (−) group, *p < 0.05, ***p < 0.001 (Student’s unpaired *t*-test or Mann–Whitney U test according to the data distribution). (**e, f**) The representative images of NHCF-A cells that differentiated into αSMA-positive myofibroblasts (**e**) and the quantification of αSMA-positive area (**f**). NHCF-A cells were passaged more than five times and then cultured with or without 50 mM LiCl for 48 h. Red: αSMA (myofibroblasts), blue: DAPI (nuclei). Scale bar, 100 μm. αSMA-positive area is normalized by DAPI count (a.u.); n = 3–4/group. Error bar, mean (SD). *p < 0.05 (Student’s unpaired *t*-test).

To further test our hypothesis that primary cilia protect against fibrosis formation, we used lithium chloride (LiCl), a well-known compound to elongate the primary cilia in various cell types^50,51^, and widely used as a mood stabilizer in clinical practice^52^. Consistent with the previous reports, LiCl elongated the primary cilia in NHCF-A cells (Fig. 7a). LiCl strongly suppressed the gene expression of ECM genes induced by TGF-β1 in NHCF-A cells (Fig. 7b–d). Furthermore, NHCF-A cells that had differentiated into myofibroblasts following long culture periods (> eight to 10 passages) dedifferentiated into αSMA-negative cells upon treatment with LiCl (Fig. 7e,f).

Our data suggest that preserving primary cilia by either inhibiting its resorption mechanism or increasing its length can blunt the profibrotic response of atrial fibroblasts to multiple profibrotic stimuli and that targeting the primary cilia may form a therapeutic approach to disarm atrial fibrosis in AF.

## Discussion

We revealed a novel mechanism underlying the formation of atrial fibrosis in AF via loss of the primary cilia in atrial fibroblasts. Furthermore, we demonstrated that preserving the primary cilia in atrial fibroblasts from AF patients considerably blunted their profibrotic capacity in response to TGF-β1. Importantly, this approach was also shown to be efficacious in the profibrotic response of the fibroblasts induced by angiotensin II. These data suggest that our approach to regulating fibroblast activity by modifying their sensing mechanism via primary cilia rather than targeting a single profibrotic pathway has the potential to control atrial fibrosis formation as a whole in AF. This approach may be of importance when considering the multifactorial processes in fibrosis formation^22^. As ECM is a highly dynamic structure that is constantly metabolized by fibroblasts, targeting the primary cilia of atrial fibroblasts may halt or even reverse the atrial fibrosis in AF.

### Role of primary cilia in fibrosis formation

Loss or dysfunction of the primary cilium has been implicated in fibrosis formation in various tissues^53,54^. For instance, hepatic periportal fibrosis develops in the *Ift88*^Orpk^ mouse^55^, a congenital model of impaired ciliogenesis. Loss of *Ift88* in cardiac endothelial cells increases ECM production during the development of the aortic valve^56^. During the valve development, premature loss of primary cilia due to a missense mutation of a ciliary gene *DZIP1* or by *IFT88* conditional knockout in the valve interstitial cells induces an enhanced ECM synthesis, resulting in dysmorphic leaflets and, eventually, in mitral valve prolapse in adult^56,57^. Moreover, mutations of the ciliary proteins polycystin-1 and 2 (*PKD1 and PKD2*), the main genes causing polycystic kidney disease, results in major pathological changes, including interstitial inflammation and renal fibrosis^58–60^, where TGFβ-Smad signaling is enhanced^61^. Recently, loss of *pkd1* in activated fibroblasts was shown to attenuate TGF-β1 signaling and suppress ECM production following myocardial injury in mice^42^. This seemingly contradicting observation may lie in the biphasic mechanism of the transition from fibroblasts to myofibroblasts via primary cilia; once the critical signaling is initiated in epithelial cells and fibroblasts through intact primary cilia, shortening or loss of primary cilia facilitates the transition and sustains the activity of myofibroblasts^62^. Therefore, deleting *pkd1* in activated fibroblasts isolated from neonatal or relatively young mice possibly impairs the initial signaling required to differentiate fibroblasts into myofibroblasts. The fibroblasts isolated from relatively aged AF patients, on the other hand, may long have been exposed to the pathological stimuli, which may have resulted in a systematic shortening of primary cilia over time, thus making these cells prone to eliciting profibrotic responses to various profibrotic stimuli. Further studies focusing on the spatiotemporal regulation of fibroblasts into myofibroblasts’ transition via primary cilia in AF pathology will explain these ostensibly inconsistent cellular responses.

We recently showed that culturing cardiomyocytes with fibroblasts lacking primary cilia reduced the speed of activation wave propagation across the monolayer. This was mediated by extracellular matrix remodeling rather than changes to cardiomyocyte action potential properties such as depolarized resting membranes, likely precluding oxidative stress-mediated electrical remodeling or cell death^63^.

Furthermore, the shortened primary cilia observed in the AF fibroblasts may alter the Hedgehog signaling, whose function is largely unknown in AF pathology. Hedgehog signaling is crucial for cell differentiation and tissue formation, and primary cilia are central transducers of the Hedgehog signaling pathway. Hedgehog dysregulation has been implicated in pulmonary fibrosis^64^. Activation of the Hedgehog signaling leads to effects similar to those observed, namely ECM accumulation and myofibroblast differentiation. Further studies are, however, required to illustrate the involvement of this pathway in AF.

### AURKA and NEDD9 axis: a potential druggable target in AF

We demonstrated that PHA successfully restored the formation of primary cilia in human atrial fibroblasts. Furthermore, PHA suppressed the profibrotic response of atrial fibroblasts from AF patients, and its mechanism of action was cilia-dependent. Alisertib (MLN8237) is an oral selective inhibitor of AURKA, under investigation and developed for the treatment of malignancies^65^. Hence, AURKA is a druggable target. At this stage, specific chemotherapy is not indicated for AF patients. However, as *AURKA* and *NEDD9* are highly expressed in the left atrial tissue of AF patients, and Alisertib has been described to have no clinically relevant effects on cardiac repolarization or ECG parameters^66^, targeting NEDD9/AURKA/HDAC6 axis is attractive as a novel treatment for fibrosis in AF. To note, functions of *AURKA* extend beyond cilia disassembly to regulating mitochondrial function and DNA repair^67,68^. Accordingly, it is worthwhile to further examine the contribution of other downstream pathways to AF pathogenesis.

Interestingly, *NEDD9,* which stabilizes AURKA^38^, is highly expressed in endothelial cells and fibroblasts in the heart (The Single Cell Type Atlas, http://www.proteinatlas.org). This expression pattern of *NEDD9* may limit the activation of AURKA/HDAC6 and subsequent disassembly of primary cilia to those cell types in the heart. Indeed, primary cilia are primarily formed in the cells that exited from the cell cycle^69^, such as cardiomyocytes, and their formation is suppressed in actively proliferating cells. Therefore, this cilia biology may allow us only to target the cell population that is largely responsible for fibrosis formation. In either case, more detailed studies focusing on the impact of selective AURKA inhibitors on each cardiac cell population will be required before any clinical application.

### Potential therapeutic role of lithium in AF patients

We demonstrated that LiCl, well-known to elongate primary cilia in several cell types^50,51^, strongly inhibited TGF-β1 induced-upregulation of ECM genes in NHCF-A cells. Furthermore, LiCl induced dedifferentiation of αSMA-expressing myofibroblasts into αSMA-negative fibroblasts. This suggests that the elongation of primary cilia by LiCl may abrogate existing atrial fibrosis in AF patients if the same effect occurs in-vivo. Lithium is reported to promote cilia length by activating α-tubulin acetyltransferase-1 and inhibiting GSK-3β^70^; however, it has pleiotropic effects on various cellular signaling^71^, and its exact mechanism of action is not fully understood. From a clinical point of view, lithium may be beneficial for AF patients; lithium at low doses seems to have an antiarrhythmic effect^72–74^, and, as a potent inhibitor of GSK-3β^75^, it inhibits myocardial apoptosis in diabetic myocardium^76^. However, like most antiarrhythmic drugs, lithium intoxication may result in unfavorable electrocardiographic and proarrhythmic changes^77^.

### Limitations

In this study, we studied cardiac tissues from patients who underwent CABG and/or aortic valve replacement without a history of AF as control. The control tissue is, therefore, not of healthy controls. Loss of the primary cilia in fibroblasts from AF patients may also arise from different underlying cardiac diseases between the two biological groups. When we take into account the frequent coexistence of AF with other cardiac conditions such as coronary artery disease^78^ and heart failure^3^, as well as the unfeasibility of using healthy heart tissue, we argue that using patients with other cardiac diseases but without AF may be a comparable control in the context of AF to reveal the impact of AF on the cellular physiology of cardiac cells.

A limitation of this study is that the left atrial appendage (LAA) was utilized as a surrogate for left atrial tissue. This is due to the feasibility of obtaining it from live patients during surgery, as was done in previous studies^14,79^. Although no study comprehensively examined how fibrotic remodeling in the left atrium corresponds to that in the LAA, proportional changes of fibrosis parameters in both tissues, as assessed by CT, predict the same outcome^80^. We posit that the findings of this study are not limited to fibroblasts residing in the LAA but extend to fibrosis elsewhere in the atrium.

Moreover, the attenuated expression of *IFT8*8 in the left atrial tissue of AF patients cannot be specifically attributed to fibroblasts. This is because IFT88 is essential to the structure of cilia, which are present on virtually all cells. Nevertheless, cardiac fibroblasts, smooth muscle, and endothelial cells more dominantly express *IFT88* than cardiomyocytes (The Single Cell Type Atlas, http://www.proteinatlas.org).

Importantly, all the experiments in this study were performed using human tissue and isolated cells from healthy donors and patients with or without AF. The design of this study precluded the demonstration of a cause (cilia loss in fibroblast) and effect (onset of AF) relation in vivo, for which interventions in *ex-vivo* organ culture or organisms modeling AF in animals such as murine AF models are warranted. Therefore, the causal relationship *in-vivo* remains uncertain. Nevertheless, we demonstrate that loss of the primary cilia in AF results in increased fibrotic activity, which can be reversed by promoting the formation of primary cilia in the atrial fibroblasts.

## Methods

### Patient population and materials

A total of 25 patients without AF (non-AF) and 26 patients with persistent AF (AF) were included in this study (Table 1). The 25 non-AF patients were from the 150 participants of the PREDICT AF study (NCT03130985), which aims to discover biomarkers both in cardiac tissue and blood to predict new-onset AF. PREDICT-AF participants underwent cardiac surgery, had a CHA_2_DS_2_-VASc stroke score of at least two, and had no history of AF^44,45^. Persistent AF patients included in this study underwent thoracoscopic ablation for AF consisting of pulmonary vein isolation and additional left atrial lines. From both non-AF and AF patients, the left atrial appendage (hereafter, left atrial tissue) was excised, swiftly washed in the calcium-free buffer (100 mM NaCl, 10 mM KCl, 5 mM MgSO_4_, 5 mM D-Glucose, 50 mM Taurine, 5 mM MOPS) and immediately snap-frozen in liquid nitrogen on-site. The snap-frozen tissues were stored at − 80 °C. Additionally, 11 AF patients (one paroxysmal, eight persistent, and two long-standing persistent AF) were included in this study (supplemental Table 2) to isolate atrial fibroblasts from their fresh left atrial tissue. The excised atrial tissue was swiftly washed in calcium-free buffer on-site and immediately brought to the laboratory on ice. This study is in accordance with the declaration of Helsinki, and all the procedures, including the use of patients’ materials, were approved by the ethics committee of the Amsterdam University Medical Centers, University of Amsterdam. All the patients enrolled in this study provided written informed consent.

### Isolation of atrial fibroblasts from the patients and cell culture

The atrial fibroblasts from non-AF and AF patients were enzymatically isolated from fresh left atrial tissues. Briefly, the fresh tissue brought to the laboratory was dissected into pieces at a size of approximately 1 mm^3^. After repeated washing followed by a pre-digestion with 1 mg/mL collagenase A (Roche, Cat#. 10103586001) and 500 μg/mL protease XXIV (Sigma, Cat#. P-8038), the tissue pieces were digested with 1.25 mg/mL collagenase A in 10 μM calcium buffer solution for one to two hours. The cell suspension was filtered by a 40 μm-cell strainer (Corning™, Cat#. 15360801) and seeded directly on culture dishes containing DMEM (Gibco, Cat#. 41966-029) supplemented with 10% FBS, 1% penicillin–streptomycin and 0.1% Gentamycin. Twenty-four hours after seeding, the culture dishes were washed twice with PBS to remove floating non-adhesive cells and replenished with fresh medium. The medium was changed every three days, and the fibroblasts were propagated for approximately two weeks before they were used for respective assays without passaging. For RNA interference, cells were cultured with the mixture of Silencer™ Negative Control (Invitrogen™, Cat#. AM4611) or Silencer Select si*IFT88* (Invitrogen™, Cat#. 4392420, siRNA ID. s224871) in Lipofectamine RNAiMAX (Invitrogen™, Cat#. 13778150) for 24 h.

### Cell culture of normal heart cardiac fibroblasts from atria

Normal Heart Cardiac Fibroblasts from Atria (hereafter NHCF-A cells) were purchased from LONZA (Cat#. CC-2903) and cultured in the growth medium; FGM™-3 added with hFGF-B and insulin (Cat#. CC-4526). To knockdown *IFT88*, Silencer Negative Control (Invitrogen™, Cat#. AM4611) or Silencer Select si*IFT88* (Invitrogen™, Cat#. 4392420, siRNA ID. s224871) were delivered into cells using Lipofectamine RNAiMAX (Invitrogen™, Cat#. 13778150), incubated for 24 h. Following transfection, NHCF-A cells were cultured with DMEM containing 0.5% FBS for six hours to promote the formation of primary cilia. NHCF-A cells were then cultured for 48 h with/without profibrotic cytokines, e.g., TGF-β1 (2 ng/mL) or angiotensin II (10^−6^ M) in the presence or absence of compounds promoting cilia formation, e.g., 2 μg/mL of PHA-680632 (Selleck Chemicals, Cat#. S1454, purity; 96.62%) or 50 mM of LiCl (Merck, Cat#. L4408, purity; ≥ 99.0%). Most of our experiments used NHCF-A cells of passages three to seven. This is per the manufacturer’s protocol that assures retaining the fibroblast trait in these passages. NHCF-A cells passaged beyond the indicated passage (passages eight to 10) were used only for the purpose of testing the effect of LiCl on the dedifferentiation of αSMA-expressing NHCF-A.

### Immunohistochemistry and immunocytochemistry

The snap-frozen left atrial tissues from the patients were cryo-sectioned (5 μm in thickness) and fixed with 4% PFA. The sections were blocked using SuperBlock™ (Thermo Scientific™, Cat#. 37515) for 10 min at room temperature and incubated with anti-acetylated α-tubulin (Abcam, Cat#. 24610) and anti-vimentin antibody (Abcam, Cat#. ab92547) overnight at 4 °C. Anti-mouse IgG conjugated with Alexa-568 (Invitrogen™, Cat#. A-11004) and anti-rabbit IgG conjugated with Alexa-488 (Invitrogen™, Cat#. A-11008) were used as second antibodies. The sections were mounted with ProLong™ Gold Antifade Mountant with DAPI (Invitrogen™, P36931). The section of each patient was anonymized, and the images were captured by Leica TCS SP8 X confocal microscope. The proportion of the fibroblasts with primary cilia in the tissue was quantified blinded for study groups using ImageJ.

For immunocytochemistry, the cells were washed twice with PBS and subsequently fixed with 4% PFA for 10 min at room temperature. The cells were blocked with 5% BSA in PBS for one hour at room temperature, permeabilized with 1% Triton X-100 and incubated with anti-acetylated α-tubulin and anti-vimentin antibody or anti-α-tubulin (Abcam, Cat#. Ab11317), or anti-α-smooth muscle actin antibody (Dako, Cat#. M0851) overnight at 4 °C. The images of primary cilia were captured by Leica TCS SP8 X confocal microscope, and the images of αSMA-expressing cells were captured by Leica DM6000. Almost all the NHCF-A cells treated with TGF-β1 or passaged beyond passage seven turned into αSMA-expressing cells, resulting in difficulty in quantifying αSMA (+) cells (%). Therefore, αSMA-positive area was measured as a surrogate parameter of αSMA (+) cells (%). The proportion of αSMA-positive cells, the proportion of ciliated cells (counted visually with count tracking), and the length of primary cilia (using the straight line tool) were analyzed by ImageJ, and the anonymized sections were unblinded after analysis.

### Quantitative polymerase chain reaction (qPCR)

First, snap-frozen left atrial tissues were mechanically crushed into fine powder in liquid nitrogen (cryo-milling). Total RNA was then extracted from the cryo-milled patient samples or the cultured fibroblasts using TRIzol Reagent (Invitrogen™, Cat. No. 15596018), and 500 ng of total RNA was reverse transcribed by SuperScript II Reverse Transcriptase (Invitrogen™, Cat. No. 18064022). qPCR was performed on the platform of Light Cycler 480 using SYBR Green I Master (Roche, Cat. No. 04707516001), and the obtained data was analyzed by LinRegPCR method (Amplification efficiency: linking baseline and bias in the analysis of quantitative PCR data).

### Western blot

Proteins were extracted from cryo-milled left atrial tissues of the patients. The protein concentration of the samples was determined by Pierce BCA protein assay kit (Thermo Scientific™, Cat No. 23225). Thirty-μg of total protein was separated in the 4–20% gradient precast gel (Bio-Rad, Cat. No. 456-1094) by electrophoresis and transferred to the 0.2 μm PVDF membrane (Bio-Rad, Cat. No. 1704272). The membrane was then incubated with primary antibody solution containing anti-acetylated α-tubulin, anti-HDAC6 (Proteintech, Cat#. 12834-1-AP), anti-α-tubulin (Santa Cruz, Cat#. Sc-5286), anti-GAPDH (Fitzgerald, Cat#. 10R-G109a), and anti-IFT88 antibody (Proteintech, Cat#. 13967-1-AP). Amersham ECL HRP-conjugated Mouse or Rabbit IgG antibody (GE Healthcare, Cat#. NA-931 and Cat#. NA-934) was used as a second antibody, and the signals were detected using ECL™ Prime (GE Healthcare, Cat#. RPN2232). The density of the protein bands was quantified by densitometry of ImageJ and normalized relative to GAPDH.

### Statistics

SPSS (version 26) was used for the statistical analyses. The normality assumption was examined using a histogram and the Shapiro–Wilk test. The comparisons between the two groups were tested by unpaired Student’s *t*-test or Mann–Whitney U test according to the data distribution. Statistical significance between more than two groups was assessed by one-way ANOVA or the Kruskal–Wallis test according to the data distribution, and the *p*-values of each comparison were corrected by post-hoc analysis. The comparisons between the treatments using the fibroblasts isolated from the patients were tested by paired *t*-test or Wilcoxon signed-rank test. The comparison was regarded as significant when the *p*-value was < 0.05.

### Supplementary Information

Supplementary Information 1.

Supplementary Information 2.

## Data Availability

The data that support the findings of this study are available from the corresponding author upon reasonable request.
