# Supplementary material for: Primary cilia suppress the fibrotic activity of atrial fibroblasts from patients with atrial fibrillation in vitro
Source: Sci Rep. 2024 May 30;14:12470. doi: 10.1038/s41598-024-60298-x (PMC11139955; doi:10.1038/s41598-024-60298-x)
Supplement: Supplementary file 1 — Supplementary Information 1. [file 41598_2024_60298_MOESM1_ESM.docx]

**Supplemental Figure 1**

**Supplemental figure 1**

(**a**) Representative images of human atrial fibroblasts doubled stained with antibodies against acetylated α-tubulin (ciliary axoneme, red) and γ-tubulin (basal body, green), showing the ciliary structure as indicated by the white arrows. Scale bar, 20μm. (**b**) The proportion of vimentin-negative cells with primary cilia (%) in the left atrial tissue of the non-AF and AF patients. N=10/group. (**c-d**) The gene expression of *PIFO* (**c**) and *TCHP* (**d**) in the left atrial tissue of non-AF (N=25) and AF patients (N=26). The statistical significance was tested using the Mann-Whitney U test.

**Supplemental figure 2**


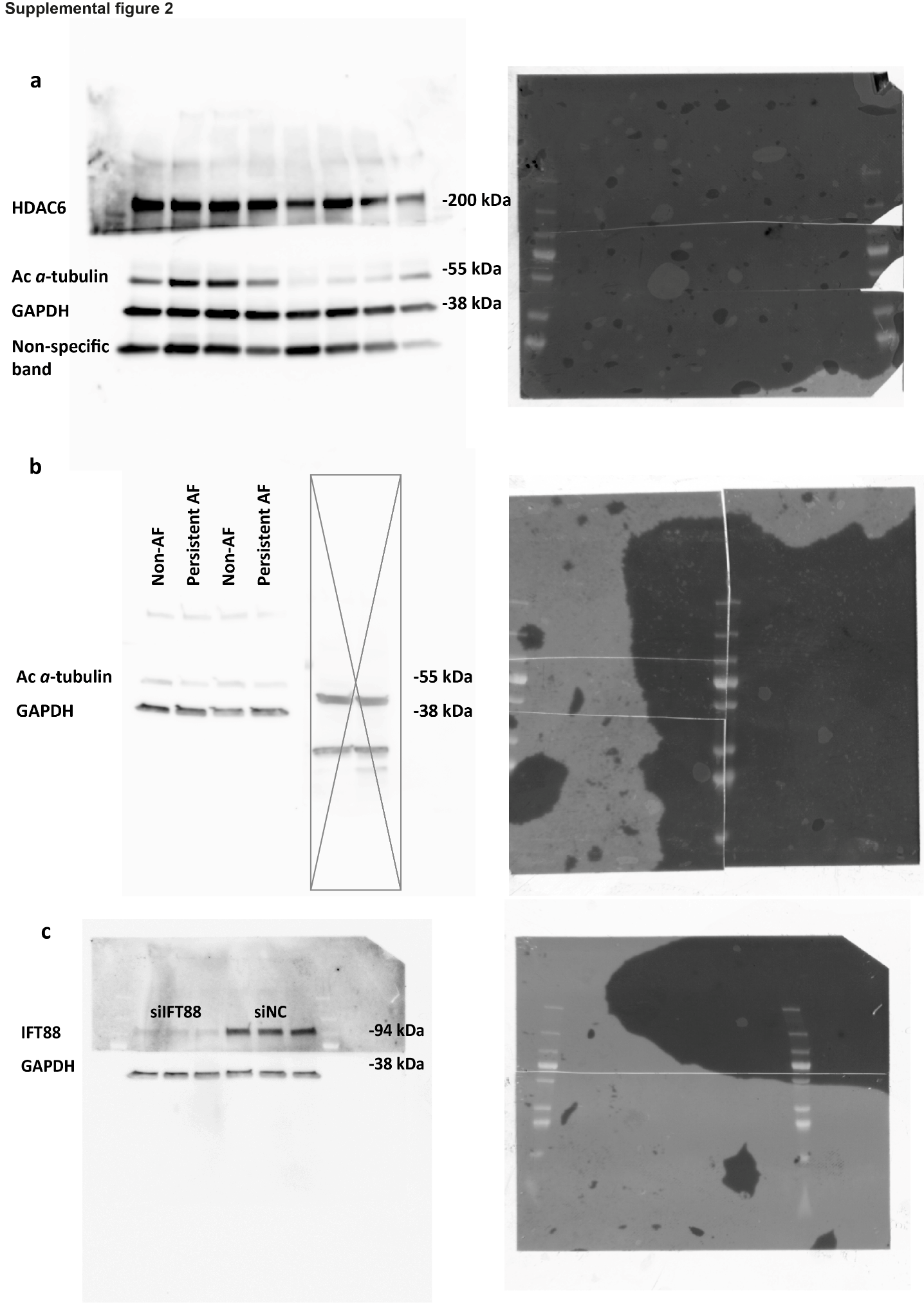


**Supplemental figure 2**

(**a, b, c**) Full-length Western Blots, including the corresponding ladders that reveal the blot edges. **(b)** The box indicates samples not relevant to the study.
