# Supplementary material for: Primary cilia suppress the fibrotic activity of atrial fibroblasts from patients with atrial fibrillation in vitro
Source: Sci Rep. 2024 May 30;14:12470. doi: 10.1038/s41598-024-60298-x (PMC11139955; doi:10.1038/s41598-024-60298-x)
Supplement: Supplementary file 2 — Supplementary Information 2. [file 41598_2024_60298_MOESM2_ESM.pdf]

Supplemental table 1. Clinical characteristics of the patients enrolled in the

	**Non-AF (n=10)**	**Persistent AF (n=10)**	***p*-value**
**Surgery type**			
VATS PVI	-	10 (100)	*NA*
CABG	10 (100)	-	*NA*
Aortic valve	1 (10)	-	*NA*
CABG+valve	1 (10)	-	*NA*
**Baseline**			
Sex, male, n(%)	10 (100)	10 (100)	*NA*
Age, years (±SD)	72.5±6.4	69.2±6.6	0.269
AF duration, years [IQ]	-	5 [1.5-9]	*NA*
Previous catheter PVI, n(%)	-	1 (10)	*NA*
BMI, kg/m^2 (±SD)	27.2±1.5	26.4±4.1	0.85
Creatinine, ml/l (±SD)	86.6±11.0	94±10.4	0.139
CHA2DS2-VASc [IQ]	2.5 [2-3.8]	2 [2-2]	0.056
Vascular disease, n(%)	10 (100)	1 (10)**	<0.001
Previous PCI, n(%)	2 (20)	0 (0)	0.456
Myocardial infarction, n(%)	4 (40)	1 (10)	0.302
Hypertension, n(%)	5 (50)	6 (60)	1
Diabetes Mellitus, n(%)	3 (30)	1 (10)	0.576
Stroke/TIA/embolus, n(%)	1 (10)	2 (20)	1
**Medication**			
NOAC/vitK antagonist, n(%)	0 (0)	10 (100)	<0.001
Antiplatelet, n(%)	9 (90)	0 (0)	<0.001
Class IA AAD, n(%)	0 (0)	0 (0)	*NA*
Class IC AAD, n(%)	0 (0)	2 (20)	0.456
Class II AAD, n(%)	9 (90)	5 (50)	0.143
Class III AAD, n(%)	0 (0)	2 (20)	0.456
Class IV AAD, n(%)	0 (0)	2 (20)	0.456
Digoxin, n(%)	0 (0)	1 (11)	0.957
ACE inhibitor, n(%)	2 (20)	3 (30)	1
Angiotensin II blocker, n(%)	2 (20)	2 (20)	1

VATS PVI, video assisted thoracoscopic pulmonary vein isolation. CABG, coronary artery bypass grafting. BMI, body mass index. PCI, percutaneous coronary intervention. TIA, transient ischemic attack. NOAC, non-vitamin K antagonist oral anticoagulants. AAD, anti-arrhythmic drugs. The categorical variables (eg. sex, CHA2DS2-VASc, the presence of comorbidities and medication) were compared by Chi-squared test. The continuous variables (eg. age, BMI and creatinine) were compared by Mann-Whitney U test.

Supplemental table 2. Clinical parameters of the patients whose atrial fibroblasts were used in this study

	**AF patients (n=10)**
**AF type**	
Paroxysmal AF	1 (10)
Persistent AF	7 (70)
Long-standing persistent AF	2 (20)
**Baseline**	
Sex, male, n(%)	8 (80)
Age, years (±SD)	64.5±7.1
AF duration, years [IQ]	2 [1.8-3.5]
Previous catheter PVI, n(%)	0 (0)
BMI, kg/m^2 (±SD)	29.5±3.1
CHA2DS2-VASc [IQ]	1.5 [0-2.3]
Vascular disease, n(%)	1 (10)
Myocardial infarction, n(%)	1 (10)
Hypertension, n(%)	3 (30)
Diabetes Mellitus, n(%)	1 (10)
Stroke/TIA/embolus, n(%)	1 (10)
**Medication**	
NOAC/vitK antagonist, n(%)	8 (80)
Class IA AAD, n(%)	0 (0)
Class IC AAD, n(%)	2 (20)
Class II AAD, n(%)	6 (60)
Class III AAD, n(%)	7 (70)
Class IV AAD, n(%)	1 (10)

PVI, pulmonary vein isolation. BMI, body mass index. TIA, transient ischemic attack. NOAC, non-vitamin K antagonist oral anticoagulants. AAD, anti-arrhythmic drugs.
